# Protein Methylation and Stress Granules: Posttranslational Remodeler or Innocent Bystander?

**DOI:** 10.4061/2011/137459

**Published:** 2011-02-24

**Authors:** Wen Xie, Robert B. Denman

**Affiliations:** ^1^Division of Hematology and Medical Oncology, Department of Medicine, Weill Medical College of Cornell University, New York, NY 1065, USA; ^2^Biochemical Molecular Neurobiology Laboratory, Department of Molecular Biology, New York State Institute for Basic Research in Developmental Disabilities, 1050 Forest Hill Road, Staten Island, NY 10314, USA

## Abstract

Stress granules contain a large number of post-translationally modified proteins, and studies have shown that these modifications serve as recruitment tags for specific proteins and even control the assembly and disassembly of the granules themselves. Work originating from our laboratory has focused on the role protein methylation plays in stress granule composition and function. We have demonstrated that both asymmetrically and symmetrically dimethylated proteins are core constituents of stress granules, and we have endeavored to understand when and how this occurs. Here we seek to integrate this data into a framework consisting of the currently known post-translational modifications affecting stress granules to produce a model of stress granule dynamics that, in turn, may serve as a benchmark for understanding and predicting how post-translational modifications regulate other granule types.

## 1. Introduction

**Figure 1:**
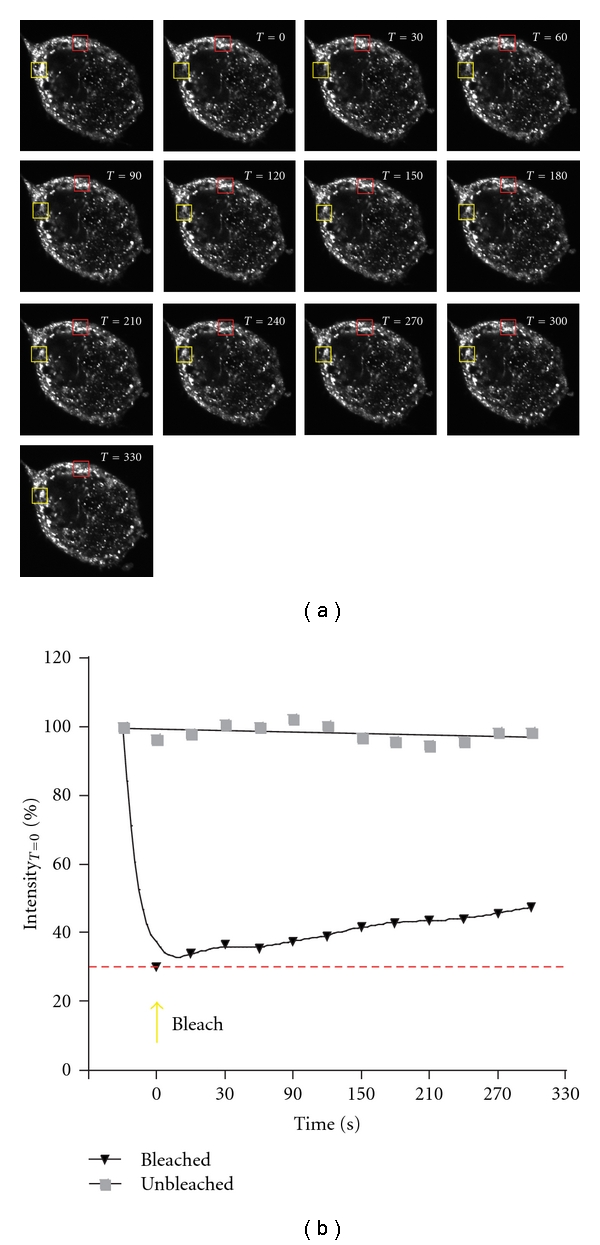
FRAP shows the dynamic exchange of FMRP between stress granules and the cytosol. (a) A representative HeLa cell treated with 1.0 mM sodium arsenite and expressing an EGFP-tagged form of the fragile X mental retardation protein (FMRP) was imaged by confocal microscopy. The region of interest (ROI) in the yellow box was photobleached to 30% of its initial intensity at time 0. The recovery of EGFP-FMRP fluorescence over the next 330 seconds within the ROI is shown in the sequentially numbered panels. A similar sized ROI shown in red that was not photobleached was quantified similarly. (b) The graph shows the fluorescence intensities in both panels as a function of time. The results were adapted from Dolzhanskaya et al. [27, Figure  9] and Dolzhanskaya et al. [20, Figure  7].

Stress granules are large, complex ribonucleoprotein particles that form in response to cellular insults such as heat shock, oxidative stress, energy deprivation, and glucose starvation [1–3]. They contain messenger RNA, small ribosomal subunits, eukaryotic initiation factors, and a host of RNA-Binding proteins. Stress granules are sites of RNA processing, and as such they are in dynamic equilibrium with other components of the cytosol and even the nucleus. For example, Kedersha et al. have shown that stress granules are reciprocally linked to polyribosome formation [4]. Additionally, certain RNA-Binding proteins have been shown to shuttle between stress granules and processing bodies [5]. Finally, fluorescence recovery after photobleaching studies (FRAP) have demonstrated that the components in stress granules can be replaced by their soluble counterparts in the cytosol to varying degrees and rates [5–7] leading one to the view that these entities can be modulated by a variety of forces that affect their composition (Figure 1).

Posttranslational modifications are known to affect protein-protein interactions between messenger ribonucleoprotein particles (mRNPs) [8, 9], so it is not surprising that they also play important roles in the assembly and remodeling of stress granules. In fact, the classic trigger for stress granule formation arising from heat shock or oxidative stress involves the phosphorylation of eukaryotic initiation factor 2*α* (eIF2*α*) [10–12]. In addition, other phosphorylation/dephosphorylation events also have been shown to affect stress granule composition, assembly, and disassembly [13–15].

Recent work has expanded the list of Posttranslational modifications that can affect stress granule composition and dynamics. These include the recruitment of calreticulin to stress granules by its arginylation [16], the dependence of histone deacetylase 6 (HDAC6) activity for stress granule formation [17], and the discovery that inhibiting the ubiquitin-proteasome system leads to stress granule disassembly even in the continued presence of eIF2*α* phosphorylation [18]. Finally, studies originating from our laboratory have demonstrated that stress granules contain methylarginine-containing proteins [19, 20]. In this paper we will examine the results implicating a role for protein methylation in stress granule function. We will then endeavor to integrate protein methylation into a general scheme detailing how Posttranslational modifiers act on stress granules and possibly other granules as well.

## 2. RNA-Binding Proteins Containing RG Rich Regions Are Methylated on Specific Arginine Residues

RNA-Binding proteins are associated with virtually all aspects of cellular RNA metabolism. RNA Binding, however, is mediated by a relatively small number of protein domains whose type, repeat-number, and sequential order within a protein dictate the RNA-Binding properties of that protein. The structure and composition of many RNA-Binding domains such as the RRM (RNA recognition motif) are well defined. Others, however, are more heterogeneous. An RNA-Binding domain that has not been well defined is the RGG box [21]. Nevertheless, it is clear that RNA-Binding proteins that harbor RGG boxes, or more precisely, RG-rich regions, play important roles in many aspects of RNA processing. They do so in two interrelated ways. First, glycine-flanked arginine residues within RGG repeat motifs serve as target sites for Type I protein arginine methyltransferases (PRMTs), and methylation of specific arginine residues can have varied effects on a protein's RNA-Binding activity, its ability to interact with other proteins and its intracellular localization [22, 23]. Second, alternative splicing, in and around RG-rich domains has been shown to modulate both nucleic acid binding [24, 25] and protein methylation [26].

## 3. Asymmetrically Dimethylated RNA-Binding Proteins Are Found in SGs

The fragile X mental retardation protein (FMRP) contains an RG-rich region, which is encoded by alternatively spliced exon 15 [29]. Our discovery that this region could be methylated *in vitro* [30] led us to wonder if the methylation had any functional consequences on FMRP biology. Specifically, we were interested in testing whether methylation may be a trigger for the incorporation or release of FMRP from neuronal granules. Since biochemical studies pointed to the fact that methylation was required for FMRP to interact with its paralog, FXR1P [19], we wished to investigate whether the FMRP/FXR1P content was altered in granules when cells were hypomethylated. Initial immunostaining studies showed that endogenous FMRP forms small cytoplasmic granules [19] that were present in a wide variety of cultured cells [31]. These granules were unique because they did not colocalize with other known granule markers, nor did they contain mRNA or methylated proteins. In addition, when HeLa cells were treated with adenosine 2′,3′ dialdehyde (AdOx), a general methylation inhibitor, the number of cells harboring such granules increased [19]. 

**Figure 2:**
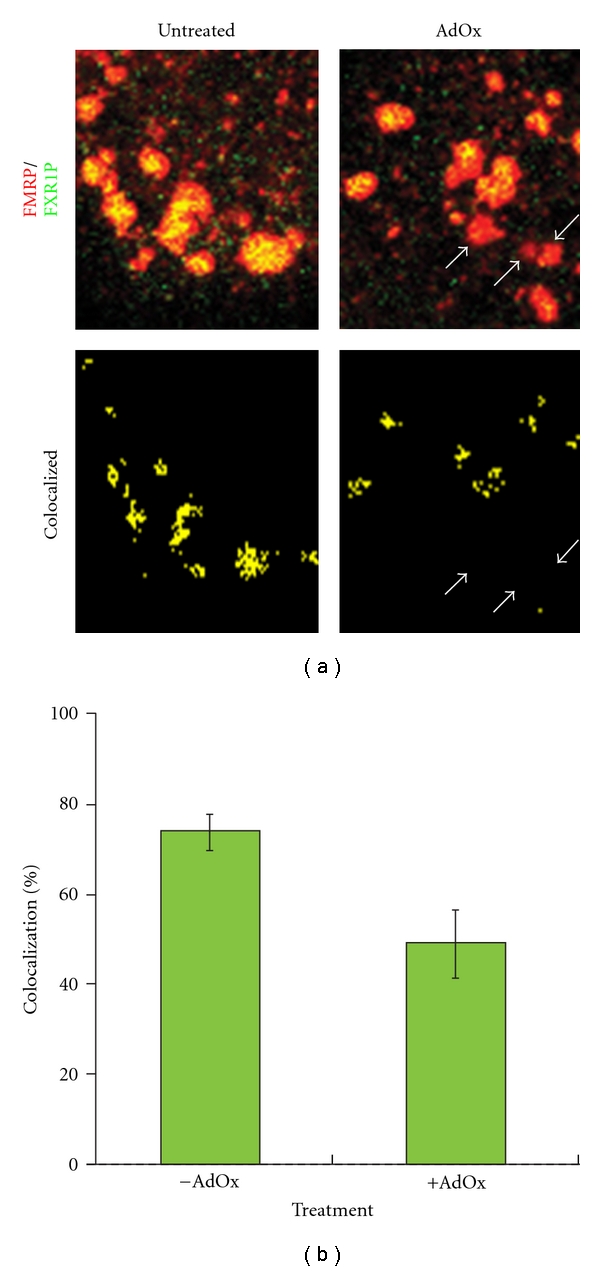
Inhibiting cellular methylation alters the composition of FMRP-containing stress granules. (a) HeLa cells were either treated for 24 hrs with 10 *μ*M AdOx to reduce cellular methylation or left untreated. Subsequently, the cells were treated with 0.5 mM sodium arsenite for 20 minutes to induce the formation of stress granules and then immunostained with antibodies, which detect FMRP (red) and its paralog FXR1P (green). The cells were then imaged by confocal microscopy. The upper panels show a region within a cell exhibiting a robust amount of stress granules. FMRP and FXR1P colocalizing stress granules (lower panels) were computed using Image J by multiplying the red and green fluorescence intensities according to Kayali et al. [28]. The arrows mark stress granules in the AdOx-treated cell which are devoid of FXR1P. (b) The graph quantifies the extent of FMRP/FXR1P colocalization as a function of AdOx treatment for more than 800 stress granules for each treatment (*P* <  .01, ANOVA). All of the results have been adapted from Dolzhankaya et al. [19, Figure  6 and Table  1].

As a control for these studies we examined the cellular methylation state and its effect upon FMRP in stress granules. Here we clearly demonstrated that upon cellular hypomethylation a portion of the FMRP is lost from stress granules as its colocalization with stress granule markers such as TIA1 and PABP decreases. This change correlated with a decrease of FXR1P in FMRP-containing stress granules (Figure 2), a loss of FMRP in FXR1P immunoprecipitates, and with an increased association of FMRP with smaller cytoplasmic granules [19].

**Figure 3:**
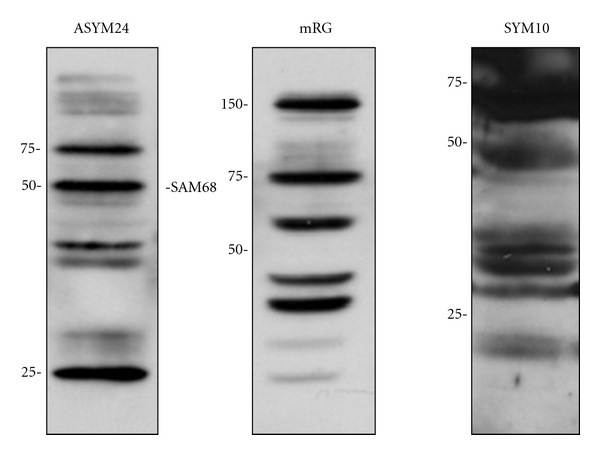
Dimethylarginine antibodies detect subsets of cellular proteins. Total mouse brain proteins (40 *μ*g) were resolved by SDS-PAGE and probed with asymmetric dimethylarginine-specific rabbit polyclonal antibodies (ASYM24) or (mRG) or the symmetric dimethylarginine-specific rabbit polyclonal antibody (SYM10). Similar results are observed in cultured cells. The effect of AdOx on cellular protein methylation and the dose-dependent decrease following AdOx treatment using these antibodies can be found in Dolzhanskaya et al. [27, Figures  10 and 13], [20, Figures  8 and 11], and [19, Figure  S1].

To confirm that AdOx treatment truly resulted in decreased cellular methylation we conducted Western blotting [19, 27] and immunostaining studies [19] using two antibodies that detect subsets of asymmetrically dimethylated (aDMA) proteins (Figure 3). Both assays showed a dose-dependent decrease in asymmetrically dimethylated proteins. However, the decreases for individual proteins were not uniform as they are most likely linked to the rate of protein turnover [32]. In keeping with this observation, stress granules from AdOx-treated cells stain positive for asymmetrically dimethylated proteins, although much more weakly so than nontreated cells [20].

Because the antibodies used to detect asymmetrically dimethylated proteins are incompletely characterized and validated [33, 34] the identities of most of the methylated proteins that they detect in stress granules are not wellknown. Nevertheless, over the years a list of experimentally methylated RNA-Binding proteins that are recruited to stress granules upon a variety of cellular insults has grown (Table 1). These 14 proteins demonstrate that changes in cellular methylation have the potential for significantly modulating the composition and the function of stress granules. For example, the cold inducible response protein, CIRP, is methylated on arginines 94, 105, and 116 of its C-terminal RG-rich region. AdOx treatment completely blocks CIRP's nuclear export and its subsequent recruitment into stress granules [1]. On the other hand, as mentioned above, AdOx treatment reduces, but does not completely block, the recruitment of FMRP into stress granules.

## 4. The limitations of AdOx Studies

Is methylation obligate for stress granule formation? This turns out to be a much more difficult question to answer than one might think. The reason seems to be a function of the stability of the products of N-methylation reactions and the general dearth of cellular demethylases. As mentioned above, inhibiting cellular methylation with AdOx results in an increase in hypomethylated proteins, which are readily observed by comparing the patterns of AdOx-treated versus nontreated protein extracts by Western blotting with anti-methylarginine antibodies [1, 19] or by *in vitro* methylation [35]. Nevertheless, Chen et al. have shown that the changes in methylation upon AdOx treatment are specifically associated with new protein synthesis. Most cellular methylation does not change. That is, once a protein is methylated it generally remains so until it is turned over [32]. Because of the background of methylated and partially methylated proteins in cells treated with AdOx one would need a means of monitoring both stress granule formation and the methylation status of each methylated protein that associates with stress granules to determine whether methylation plays a role in stress granule assembly. Currently, the lack of antibodies that specifically recognize only the methylated or non-methylated forms of particular RNA-Binding proteins precludes attacking the problem in this manner.

To try to circumvent this requirement we sought a paradigm in which we could monitor the recruitment of a newly synthesized methylatable protein into stress granules in the presence of AdOx. We chose FMRP because it has been demonstrated that its overexpression is sufficient for forming stress granules in the absence of cellular stressors and that this effect is mediated by its methylatable RG-rich domain [36]. Thus, we treated HeLa cells with AdOx and subsequently transfected them with a plasmid that produces EGFP-FMRP in its continuous presence. We determined that AdOx treatment had no effect on the expression efficiency of EGFP-FMRP or its ability to make cytoplasmic granules that sometimes contained other stress granule markers [20]. However, two caveats prevent us from definitively concluding that protein methylation is not absolutely required for stress granule formation. First we noted that unlike endogenous FMRP, EGFP-FMRP granules contained asymmetrically that dimethylated proteins even in the presence of AdOx [20], and our experimental protocol does not allow us to distinguish whether EGFP-FMRP recruits endogenous methylated protein to form stress granules or vice versa. Second, we have not demonstrated whether or not EGFP-FMRP is methylated in the presence of AdOx. Thus, the question of whether methylation is required for stress granule formation will necessitate further study.

## 5. Are the Proteins Found in Stress Granules Methylated before or after Stress Granule Formation?

Other important questions that have yet to be addressed are where and when are the methylated proteins found in stress granules methylated? More specifically, is there any evidence that protein arginine methyltransferases are associated with stress granules? The importance of this question cannot be underestimated. If proteins cannot be methylated, dimethylated, or hyper-/hypomethylated while, they are in stress granules, it is extremely unlikely that protein methylation would play a direct role in stress granule remodeling.

**Figure 4:**
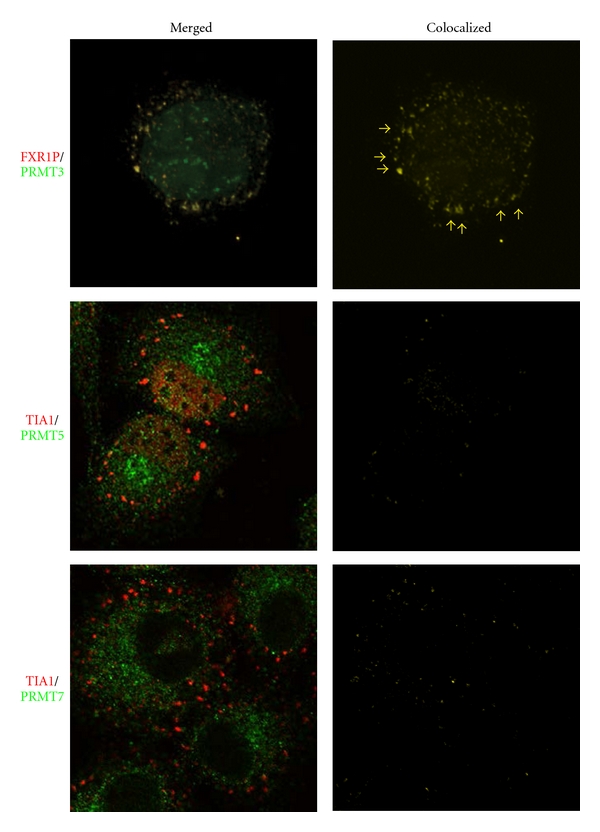
The class I protein arginine methyltransferase, PRMT3, colocalizes with stress granules. HeLa cells were treated with 1.0 mM sodium arsenite for 20 minutes and subsequently immunostained with antibodies that detect the stress granule markers, FXR1P or TIA1 (red), and ones that recognize PRMT3, PRMT5, and PRMT7 (green). The panels show a representative cell for each of the staining. Colocalized PRMT3/FXR1P granules are marked with yellow arrows. Colocalization analysis performed as described in Figure 2 shows that PRMT3 is a constituent of FXR1P-containing stress granules. In contrast, the majority of PRMT5 is found in the nucleus, and the fraction that is cytoplasmic is found in much smaller granules that sometimes associate, but do not completely overlap, with TIA1-containing stress granules. Similar results occur with PRMT7, which appears to be strictly cytoplasmic in the presence of arsenite. The results have been adapted from Dolzhanskaya et al. [27, Figure  17].

Because of its known association with ribosomes [37], we were particularly attracted by the possibility that PRMT3 might be found in stress granules. Therefore, we measured the subcellular distribution of PRMT3 in the presence and absence of arsenite treatment by confocal immunofluorescence microscopy using endogenous FXR1P as a stress granule marker (Figure 4). We found that in the presence of arsenite PRMT3 was found in perinuclear cytoplasmic granules that colocalized with FXR1P-containing stress granules. Interestingly, pretreatment of the cells with AdOx resulted in the relocation of most of PRMT3 to the nucleus, effectively eliminating the colocalization (not shown). The basis of this effect is currently unknown. In contrast, we found that neither class II PRMT (PRMT5 nor PRMT7) colocalized with TIA1-containing stress granules. These data support the hypothesis that asymmetric dimethylation, but not symmetric dimethylation, may be modulated directly on stress granules. However, additional experiments, for example, Förster resonance energy transfer (FRET) studies, will be required to confirm the colocalization of PRMT3 in stress granules.

## 6. A Link between Stress Granules, Symmetric Dimethylation, and Splicing?

**Figure 5:**
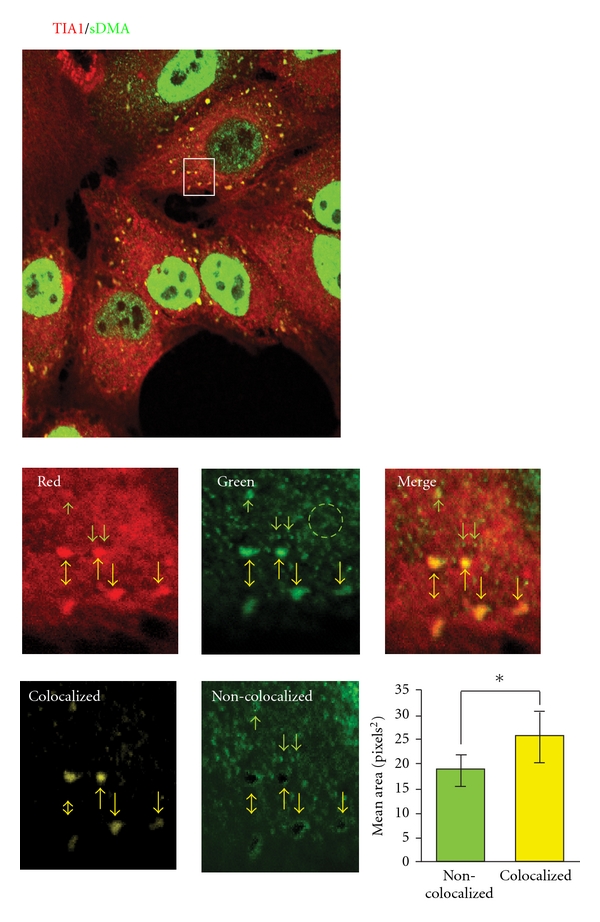
Stress granules contain symmetric dimethylarginine-containing proteins. HeLa cells were treated with 1.0 mM sodium arsenite for 20 minutes and subsequently immunostained with an antibody that detects the stress granule marker, TIA1 (red), and one that recognizes a subset of symmetrically dimethylarginine-containing proteins, SYM10 (green). The upper panel shows a confocal image of the stress granule containing cells. A magnified view of the boxed region is shown in the second set of panels. It is evident from the data that there are at least three types of granules that SYM10 recognize: large stress granules that colocalize with the TIA1 marker (yellow arrows), much smaller, non-colocalizing foci (green arrows), and a diffuse lattice or network (green circle). Colocalization analyses performed as described in Figure 2 clearly show that only the stress granules contain TIA1 and symmetrically dimethylated proteins. This was confirmed by subtracting the colocalized image from the green image; note the vacant black holes where the stress granules are. The graph quantifies the granule sizes of the colocalized and non-colocalized granules. The results were adapted from Dolzhanskaya et al. [27, Figure  14] and [20, Figure  12].

Although we found no evidence for the association of stress granules with class II protein arginine methyltransferases we still endeavored to ascertain whether stress granules contained symmetrically dimethylated (sDMA) proteins. To this end we treated HeLa cells with arsenite and then immunostained them with antibodies that recognize TIA1 and a subset of all symmetrically dimethylated proteins. We observed significant colocalization, indicating that stress granules unequivocally harbor symmetrically dimethylated proteins (Figure 5) [20].

The antibody (SYM10) that was used to detect symmetrically dimethylated arginine residues was raised to the peptide R^sDMA^GR^sDMA^GR^sDMA^GR^sDMA^G [38]. In fact, Boisvert et al. detected more than 29 potential symmetrically dimethylated proteins in immunoprecipitation reactions using this antibody [34] (Figure 3). Most of the identified proteins were associated directly or indirectly with pre-mRNA splicing. Of particular interest here were the core spliceosomal proteins (SmB/B′, SmD1/D3, and U6 small nuclear RNA-associated Sm-like proteins Lsm4 and Lsm8), which have been shown to contain symmetric dimethylarginine [39] and are known epitopes for the SYM10 antibody [38]. 

**Figure 6:**
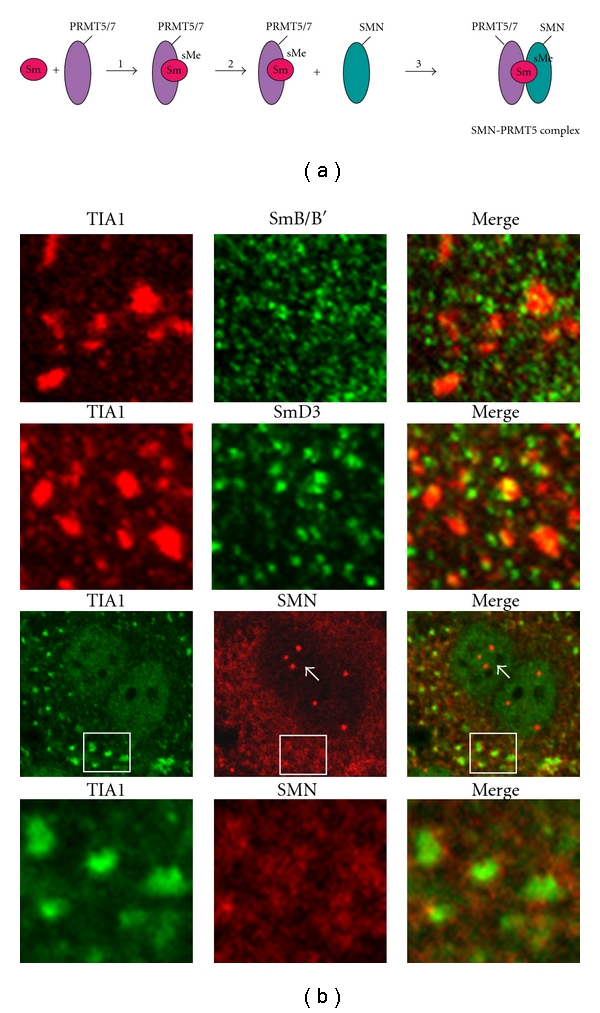
Major spliceosomal proteins do not colocalize with TIA1-containing stress granules. (a) Formation of the SMN-PRMT5 complex, which acts as the scaffold for spliceosome assembly, occurs via the binding of Sm proteins to active PRMT5 (step  1). Subsequently, Sm proteins are symmetrically dimethylated (step  2), and the resulting complex binds to the SMN complex (step  3). (b) HeLa cells were treated for 20 minutes with 1.0 mM sodium arsenite to induce stress granule formation and then immunostained with antibodies that recognize TIA1, and SmB/B′, or SmD3, or SMN. The cells were visualized by confocal microscopy. Magnified views of representative stress granule fields in the cells immunostained with the Sm antibodies are shown, highlighting the lack of colocalization between TIA1 and those proteins. For the SMN immunostaining an entire cell is shown (third panel) to demonstrate that the SMN antibody recognizes nuclear Gem bodies (white arrows) as it should [53]. A magnified view of one of the granule fields is shown in the fourth panel, highlighting the lack of colocalization between TIA1 and SMN. The results have been adapted from Dolzhanskaya et al. [27, Figures  15 and 16].

Previous studies have shown that a large number of stress granule constituents also play a role in splicing (Table 2). However, since many of these proteins also have alternative functions, for example, as translational regulators, an operational link between stress granule function and splicing has not been clearly established. For example, the exon-junction complex (EJC) protein, MLN51, is recruited to stress granules upon oxidative stress, but other minimal constituents of the EJC (MAGOH, Y14 and EIF4AIII) are not. Thus, it is unlikely that EJC function would be relevant for stress granule biology. Nevertheless, our data showing symmetrically that dimethylated proteins were associated with stress granules suggested a potential functional link might occur between stress granules and the spliceosome. To address this question we immunostained arsenite-treated HeLa cells with core components of the spliceosome and its precursor, the SMN-PRMT5 complex. We found that neither the major spliceosomal proteins anti-SYM10 recognizes, that is, SmD3 and SmB/B′, nor SMN-PRMT5 complex components, PRMT5 and SMN, were associated with stress granules (Figures 4 and 6). Thus, the identities of the symmetrically dimethylated proteins that are present in stress granules must still be determined. More importantly, however, the data rule out the possibility that stress granules function in spliceosomal chaperoning or spliceosomal triage.

## 7. Modeling the Effects of Posttranslational Modifications on Stress Granule Dynamics

**Figure 7:**
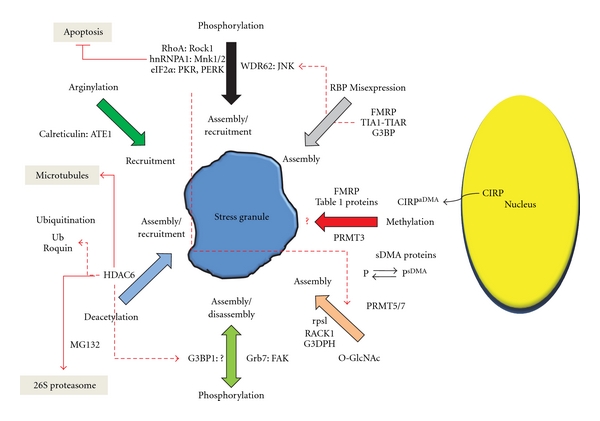
Posttranslational modifications regulate all aspects of stress granule dynamics and exhibit a complex and often hierarchical interplay. The major Posttranslational modifications that occur to stress granules are highlighted along with the modified target proteins and the enzymes that modify them. Effects of each modification on stress granule assembly, disassembly, and recruitment are described in detail in the text. Red dotted arrows show the interplay between different types of Posttranslational modifications. Red solid lines link stress granule modulation with other cellular processes (shaded boxes).

While our understanding of the mechanisms by which Posttranslational modifications regulate and remodel stress granules is incomplete, it is nevertheless clear that they do so in an intricate and often hierarchical way (Figure 7). 

Of all the Posttranslational modifications affecting stress granules, phosphorylation, by far, exhibits the most varied effects and has the largest repertoire of affected target proteins. Phosphorylation can directly trigger stress granule assembly via the eIF2*α* pathway [12], or this can occur by the dephosphorylation of Ras-GTPase-activating protein SH3 domain binding protein 1 (G3BP1) [54]. In addition, phosphorylation can have a direct bearing on the composition of stress granules through recruitment or remodeling. For example, cytosolic phosphorylation of mRNA-bound hnRNPA1 by MAP kinase interacting serine/threonine kinase (Mnk1/2) results in its recruitment into pre-existing stress granules [14]. Similarly, phosphorylation of the Ras homolog gene A (RhoA) by Rho-associated coil-coil containing protein kinase (ROCK1) leads to its recruitment into pre-existing stress granules; however, in this case active ROCK1 is also recruited into stress granules, and this action prevents cellular apoptosis [15]. Likewise, the scaffold protein WDR62 can recruit active c-jun N-terminal kinase (JNK) into stress granules and P-bodies [55], although the target(s) of the kinase within these mRNPs or its function have yet to be elucidated. Finally, phosphorylation also can affect stress granule disassembly. Recruitment of active focal adhesion kinase (FAK) to stress granules results in the phosphorylation of growth receptor bound protein 7 (Grb7) causing it is dissociation from the RNA-Binding protein HuR and consequently its release from stress granules [13]. This remodeling event is crucial for the disassembly of nascent stress granules following stress.

Deacetylation can also have wide-ranging effects on stress granule assembly and recruitment; however, unlike phosphorylation, in which a number of different kinases affect specific targets and effect specific functions, the effects of deacetylation flow through a single multifunctional enzyme, histone deacetylase 6 (HDAC6) [17]. Thus, HDAC6's deacetylase domains promote stress granule formation via their ability to bind dephosphorylated G3BP1; however, deacetylase activity is not required to mediate this effect. Nevertheless, HDAC6 activity is required for stress granule formation since mouse embryonic fibroblasts (MEFs) lacking wild-type HDAC6 are unable to do so. Furthermore, HDAC6's zinc finger ubiquitin binding domain is necessary for the recruitment of ubiquitinated proteins into stress granules, ultimately connecting stress granule dynamics to the ubiquitin-proteasome system and the ubiquitin conjugating system. Finally, HDAC6's ability to interact with microtubules links a functional microtubule network to stress granule formation.

O-linked N-acetylglucosamine modification (O-GlcNAc) has also been associated with stress assembly [56]. This modification, which occurs to a number of stress granule incorporated proteins most notably ribosomal proteins (rpsl), likely assists in the aggregation phase of stress granule formation as studies have shown that eIF2*α* phosphorylation is simultaneously required.

Other less well-studied Posttranslational modifications can also cause the modified proteins to be recruited into stress granules. Arginylation of calreticulin by the cytosolic enzyme arginine tRNA protein transferase (ATE1) following its stress-induced exit from the endoplasmic reticulum leads efficient incorporation into pre-existing stress granules [16]. Likewise, ubiquitinated proteins can also be incorporated into stress granules, following transient proteasome inhibition with MG132 as can the ubiquitin ligase, Roquin [57]. However, the role these proteins play in stress granule function is unknown.

Finally, the level of expression of various RNA-Binding proteins (RBPs) may influence their Posttranslational modification(s) and indirectly affect stress granule dynamics. Although most work has focused on the artificial overexpression of RBPs (TIA1/TIAR, FMRP, and G3BP) comparable nonequilibrium conditions could occur during development or in particular disease states such that the expression of a particular RBP exceeds the capacity of its Posttranslational modification enzymes or vice versa. For example, it has been reported that G3BP levels are decreased in fragile X syndrome [58]. This suggests that there may be a relative increase in the ratio of phosphorylated/dephosphorylated G3BP, which potentially could affect the assembly, composition or function of stress granules in fragile X patients.

## 8. Locating the Role of Protein Methylation in the Model

How does Posttranslational protein methylation fit within the overall landscape of stress granule Posttranslational modifiers? While our knowledge of this process is still in its infancy certain tentative conclusions can be drawn. First, it is apparent from the rather large number of methylated proteins that are associated with stress granules that the potential for modulating stress granule composition is great. In this regard, protein methylation resembles phosphorylation more than arginylation. On the other hand, it appears more likely that protein methylation plays its role in stress granule recruitment and remodeling, rather than in its assembly or disassembly. This is particularly true of sDMA-modified proteins whose modification occurs in the cytosol rather than in the granules and CIRP whose methylation is required for exiting the nucleus. Nevertheless, the fact that PRMT3 may associate with stress granules holds out the possibility that direct aDMA methylation or hypermethylation may occur on certain stress granules. Interestingly, PRMT3 is unique among the family of protein arginine methyltransferases in that it contains a zinc finger binding domain [59], which may via protein-protein interactions also link it to other Posttranslational modifiers like HDAC6. It is certain to say that the role protein methylation plays in stress granule function is rife for future investigations.

## 9. Concluding Remarks: Looking to the Future

Lastly, can we make any inferences between the Posttranslational processes that regulate stress granules and those affecting other granule types? First, we should distinguish between what exactly is meant by granules types. Clearly, there are two classes of large mRNP-complexes, those that are common to all cells (polyribosomes, stress granules, and P-bodies) and those that are unique to different cells (germ granules, oocyte foci, neuronal transport granules). Regardless of the class, it has been shown that they all contain common core constituents [7, 67]. Thus, there should be some underlying similarities by which they are Posttranslationally regulated. We have been particularly intrigued by the common features of stress granules and neuronal transport granules (Table 3) which may suggest that common assembly and disassembly mechanisms apply to each. Nevertheless, their distinguishing characteristics allow the possibility of unique means of Posttranslational regulation, for example, the receptor-mediated remodeling of neuronal transport granules.

Because the various granule types that have been distinguished to date share common proteins, one might expect that those proteins which are modified prior to granule formation would have two possible fates: either they would sort identically to different granules or the modification might be used to differentially sort them to specific granule types, for example, stress granules versus P-bodies. While this has been a largely uncharted area of investigation it is interesting to note that Qi et al. have demonstrated that prolyl 4-hydroxylation of Ago2 is required for its recruitment into P-bodies but not stress granules [70]. 

In contrast, modifications that occur directly on granules would likely be unique to those granules. Furthermore, it is clear that specific proteins differentiate the various granule types and this would provide unique possibilities for Posttranslational regulation. For example, it has recently been shown that the phosphorylation of Dcp2 at S137 and the phosphorylation of Ago2 at S387 are required for their accumulation in P-bodies [71]. Additionally, the repertoire of Posttranslational modifications need not be restricted to those that modify stress granules. Future studies will undoubtedly uncover a wealth of new Posttranslationally modified granule proteins, and these data will have to be integrated into comprehensive models of how these granules are formed and how they function. Until that time we have the general outline provided by Posttranslationally modified stress granules.

## 10. Summary

Protein methylation impinges upon stress granule dynamics in a number of ways that are beginning to be, but are not yet fully, defined. Time will tell if protein methylation is a true driver of stress granule stress granule assembly, disassembly, or remodeling, or whether it simply tags along, an innocent bystander on various RNA-Binding proteins that are recruited to these granules through different mechanisms.

##  Note

All of the antibodies mentioned in this paper have been described in the authors' published works [19, 20, 26, 33] with the exception of anti-SmB/B′ (Santa Cruz, sc25372), anti-SmD3 (Sigma HPA001170), and anti-SMN (2B1), a kind gift from Dr. Gideon Dreyfuss, University of Pennsylvania.

## Figures and Tables

**Table 1:** Stress granule RNA-Binding proteins containing methylatable domains.

Protein^a^	Accession no.^b^	Methylated^c^
CIRP	AAC04895	Yes
EWS	CAA51489	Yes
FMRP	XM_010288	Yes
FXR1P	P51114	Yes
FXR2P	XP_008234	Yes
FUS	CAG33028	Yes
G3BP	CAG38772	No
hnRNPA1^d^	NP_112420	Yes
hnRNPK	CAI16021	No
hnRNPQ	NP_006363	Yes
PABP1	P11940	Yes
Rps2	NP_002943	Yes
Sam68	AAH00717	Yes
SRSF1^e^	EAW94486	Yes
TAF15^f^	EAW80124	Yes

^a^Human orthologs.

^b^Experimentally verified *in vitro* or *in vivo*.

^c^Proteins can be accessed through the NCBI website by use of their GenBank accession numbers (http://www.ncbi.nlm.nih.gov/).

^d^Isoform b.

^e^Nuclear stress granules, Isoform a.

^f^Isoform a.

**Table 2:** Splicing-related proteins found in stress granules.

Protein	Function	Reference
CUG-BP1	Exonic silencing, exon skipping	[40]
FMRP	Exon splice site enhancer	[41]
hnRNPA1	Exonic silencing, exon skipping	[42]
hnRNPK	Exonic silencing, exon skipping	[43]
hnRNPQ	Intronic splice enhancer, exon inclusion	[44]
HuR	Exonic silencing, exon skipping	[45]
MBNL1	Exon enhancing, exon inclusion	[46]
MLN51	Exon junction complex	[6]
Sam68	Exonic silencing, exon skipping	[47]
SRSF1	Exon enhancing, exon inclusion	[48]
TDP-43	Context-dependent exon skipping and exon inclusion	[49]
TIA1	Intronic splice enhancing and exon inclusion	[50]
TIAR	Intronic splice enhancing and exon inclusion	[51]
YB-1	Exon enhancing, exon inclusion spliceosome associated factor	[52]

**Table 3:** Stress granules and neuronal granules share common characteristics and common members.

General	Stress granules	Neuronal granules
Large mRNPs	[19, 60]	[61, 62]
Heterogeneous composition	[63]	[64]
Localized in cytoplasm (both soma and processes)	[63]	[65, 66]
Associated with translational regulation	[67]	[66]
Motile: Exhibit microtubule-dependent movement	[60]	[65]
Remodel in response to exogenous stimuli	[19]	[65]

Specific		
Contain FXRP family members	[19, 36]	[65, 66]
Share common RBPs (G3BP, hnRNPA1, hnRNPQ, HuR, staufen, YB1)	[67, 68]	[62, 64]
Contain small ribosomal subunit proteins	[68]	[69]
Contain eukaryotic initiation factors	[67]	[61]

Bracketed numbers are relevant references.
